# Response of spatial vegetation distribution in China to climate changes since the Last Glacial Maximum (LGM)

**DOI:** 10.1371/journal.pone.0175742

**Published:** 2017-04-20

**Authors:** Siyang Wang, Xiaoting Xu, Nawal Shrestha, Niklaus E. Zimmermann, Zhiyao Tang, Zhiheng Wang

**Affiliations:** 1 Department of Ecology and Key Laboratory for Earth Surface Processes of the Ministry of Education, College of Urban and Environmental Sciences, Peking University, Beijing, China; 2 Dynamic Macroecology, Swiss Federal Research Institute WSL, Birmensdorf, Switzerland; Institute of Tibetan Plateau Research Chinese Academy of Sciences, CHINA

## Abstract

Analyzing how climate change affects vegetation distribution is one of the central issues of global change ecology as this has important implications for the carbon budget of terrestrial vegetation. Mapping vegetation distribution under historical climate scenarios is essential for understanding the response of vegetation distribution to future climatic changes. The reconstructions of palaeovegetation based on pollen data provide a useful method to understand the relationship between climate and vegetation distribution. However, this method is limited in time and space. Here, using species distribution model (SDM) approaches, we explored the climatic determinants of contemporary vegetation distribution and reconstructed the distribution of Chinese vegetation during the Last Glacial Maximum (LGM, 18,000 ^14^C yr BP) and Middle-Holocene (MH, 6000 ^14^C yr BP). The dynamics of vegetation distribution since the LGM reconstructed by SDMs were largely consistent with those based on pollen data, suggesting that the SDM approach is a useful tool for studying historical vegetation dynamics and its response to climate change across time and space. Comparison between the modeled contemporary potential natural vegetation distribution and the observed contemporary distribution suggests that temperate deciduous forests, subtropical evergreen broadleaf forests, temperate deciduous shrublands and temperate steppe have low range fillings and are strongly influenced by human activities. In general, the Tibetan Plateau, North and Northeast China, and the areas near the 30°N in Central and Southeast China appeared to have experienced the highest turnover in vegetation due to climate change from the LGM to the present.

## Introduction

At large spatial scales, distribution of natural vegetation is primarily determined by climate [1, 2]. Studies have shown dramatic alteration of vegetation pattern due to climate change events, such as the expansion of shrubs in the tundra vegetation [3] and growth retardation of shrub and grass species due to changes in precipitation seasonality [4]. Recent GCM simulations suggest that, by the end of the 21st century, the global mean temperature may increase by 2.2°C from the mean value during 1986–2005 [5]. Such dramatic climate change will significantly influence the distribution of terrestrial vegetation in the future. Moreover, recent studies have shown that tropical forests tend to decrease the local temperature (local cooling) whereas temperate forests tend to increase it [6], which suggests that changes in the distribution of vegetation could affect the local climate through cooling and warming effects [7] and by altering carbon and water cycles regionally [8–10].

Understanding the dynamics of vegetation distribution in relation to palaeoclimate change is essential for a sound forecasting of the response of vegetation to future climate change [11, 12]. Previous long-term studies (spanning more than 1000 years) on the relationship between climate change and vegetation distribution mainly relied on pollen data. Both global [13] and regional [14–17] distributions of palaeovegetation during the Mid-Holocene (MH, 6000 yr BP) and the Last Glacial Maximum (LGM, 18,000 yr BP) have been reconstructed based on pollen data. Such reconstructions are helpful in understanding the past distribution patterns of vegetation, although they may be prone to several limitations, including limited spatial coverage in pollen surface samples, lack of coverage of small refuges [18], or difficulties in species identification. Moreover, such maps lack a vegetation-climate response function and are therefore not useful in predicting future vegetation distribution.

A suitable empirical model to project vegetation distribution into the past or the future thus needs to be built on vegetation-climate relationships. The framework of species distribution modeling (SDM) is a highly suitable method to study the association between species distributions and climate [19, 20]. SDMs use data of species occurrences and absences and corresponding environmental layers to infer environmental requirements of species and predict the distributions of species in different regions and period [19–22]. Climate also strongly affects the structure and distribution of vegetation at large spatial scales [23, 24]. Based on these strong associations, several systems for vegetation-climate classification have been developed to study vegetation distributions and their responses to climate change (e.g. [24–28]). Rubel and Kottek [24] projected the changes in climate from 1901 to 2100 and the corresponding shifts in vegetation distribution using the Köppen-Geiger climate classification. The similarity in the determinants of species and vegetation distributions suggests that the framework of SDMs can be used to predict the association between vegetation distribution and climate, as has previously been applied [29, 30]. A recent study forecasted the vegetation distribution indirectly by predicting the distribution of dominant species [31]. In another example [32, 33], the authors explored the responses of forests and savannas to future climate changes using generalized linear models.

China covers a large spectrum of vegetation types, ranging from tropical rain forests and subtropical evergreen broadleaf forests, through temperate deciduous broadleaf forests to boreal forests, and temperate and cold steppes and deserts. Based on remote sensing data, Fang et al. [33] have shown that global warming since 1980s has significantly influenced the growth and coverage of different vegetation types in China. However, the response of vegetation distribution to climate change over a longer period (e.g. since the LGM) and into the future remains poorly understood (but see [17, 34] for MH and LGM reconstruction using pollen data). One way to assess vegetation change is by means of dynamic global vegetation models, which simulate the distribution of dominant plant functional types globally based on physiological and ecosystem processes [35–37]. The advantage of such models is that they give significant details on carbon and water pools and fluxes in ecosystems, while the disadvantage is that they only map the most common (usually 6–7) plant functional types and cannot distinguish more subtle differences in vegetation.

In this study, we develop a vegetation climate model based on the contemporary 1:1,000,000 vegetation map of China and climate data sets by means of two widely-used species distribution models. Specifically, 1) we demonstrated the capability of SDMs to study the association between vegetation distribution and climate; 2) we reconstructed palaeovegetation distribution during the LGM and the MH in China and compare these results against those reconstructed by pollen data; and 3) we calculated the directions of the centroid of each vegetation type and the transformation among different vegetation since the LGM.

## Material and methods

### Modern vegetation map of China

**Fig 1:**
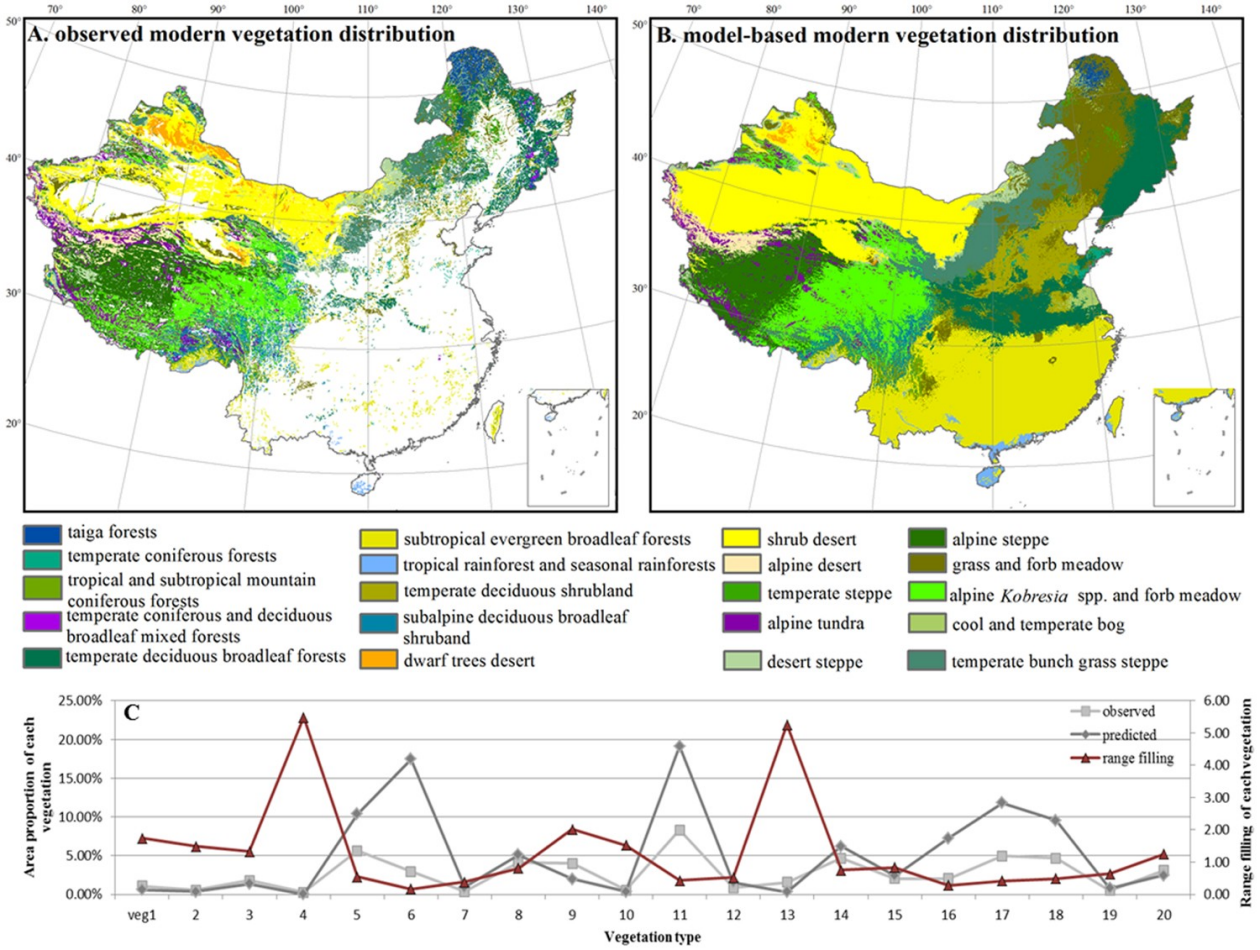
Contemporary vegetation distribution in China. (A) Observed (white area are croplands, urban areas and planted forests); (B) modeled; (C) the proportions of each vegetation type to the terrestrial area of China (observed: light gray line; modeled: dark gray line) and their range fillings calculated as the ratios between the observed and modeled distribution ranges (red line). Due to strong human disturbance, veg 5, 6, and 8 have experienced significant deforestation at the present, and therefore the proportions of observed vegetation types do not sum up to 100%.

The contemporary distribution of major vegetation types in China was derived from the *Vegetation Atlas of China 1*:*1*,*000*,*000* [38]. Therein, the natural vegetation in China is divided into 47 vegetation types and 573 vegetation formations. For further analysis, we merged 47 natural vegetation types into 20 major groups according to life-forms of dominant species in the mapped vegetation (Fig 1A). There were 57,099 polygons in total for natural vegetation, and the sizes of these polygons ranged from 0.00003 to 288,500 km^2^. In order to generate a database for modeling, we intersected the polygons with a 10 × 10 km grid, which resulted in 165,614 polygons, each contains a single vegetation type.

### Climate and topographic data

Contemporary climate data were downloaded from the WorldClim database (http://www.worldclim.org/) [39], and included monthly mean temperature and precipitation for the period of 1950–2000 and 19 biologically meaningful variables (i.e. Bio1 –Bio19, bioclimatic variables for short hereafter; see S1 Table for details of these variables). All the layers of contemporary climate have spatial resolutions of 2.5 × 2.5 arc minutes (approx. 4.5 × 4.5 km at the equator).

From the same website (http://www.worldclim.org/), we also downloaded the climate data for the Mid-Holocene (ca. 6000 yr BP) and the Last Glacial Maximum (LGM, 18,000 ^14^C yr BP) [39]. The data of past climate were statistically downscaled from the original simulations of general circulation models (GCMs) [39]. In previous studies, two GCMs have been widely used to evaluate the effects of the LGM climate on species and vegetation distributions in eastern Asia [40–43], including the Model for Interdisciplinary Research on Climate (MIROC-ESM) [44] and Community Climate System Model (CCSM) (v3 and v4) [45, 46]. Several studies have evaluated the predictions of these two GCMs and suggested that the MIROC-ESM is more realistic for eastern Asia than CCSM [40–42]. Therefore, we used the simulations of MIROC-ESM [44] for our analysis. The variables for past climate are the same as those for contemporary climate, including monthly mean temperature and precipitation and the 19 bioclimatic variables (i.e. Bio1 –Bio19, see S1 Table for details of these variables). The spatial resolution of past climate data layers is all 2.5 × 2.5 arc minutes (approx. 4.5 × 4.5 km at the equator).

In our analysis, we also used topography variables, including altitude, slope and aspect. The slope and aspect of each grid cell were calculated from the altitude data layer using ArcGIS 10.0 and the data for altitude with a spatial resolution of 30 × 30 arc seconds were downloaded from the WorldClim database (http://www.worldclim.org/).

To fit between climate and vegetation data, the contemporary and past climate data for a vegetation polygon were calculated as the averages of all cells (2.5 × 2.5 arc minutes) of the climate layers using the tool of zonal statistics in ArcGIS 10.0.

### Pollen data

Pollen data in China for the LGM and the MH were compiled from Yu et al.[17]. There are seven biomes reconstructed from fossil pollen data, which correspond to our vegetation types. In total, the dataset included 119 pollen records dated to 6000 ^14^C yr BP (±500 yr) and 37 records dated to 18000 ^14^C yr BP (±2000 yr) representing he MH and the LGM vegetation, respectively.

### Variable selection and statistical analysis

We used two SDMs to simulate the vegetation distributions, including generalized linear models (GLMs) and Maximum Entropy (MaxEnt). These two models have been widely used for analyzing ecological relationships [47, 48] and have high predictive power across large sample sizes [20]. To calibrate the models for a vegetation type, the geographic distribution of the vegetation type was used as the response variable with both presence and absence data: the polygon where this vegetation type occurs was set to be 1 and the others to 0.

Previous studies have shown that the distribution of different vegetation types may be determined by different environmental factors [49]. In a preliminary analysis, all the 19 bioclimatic variables and the three topographic variables were used to calibrate the models. However, we found strong multicollinearity between variables (S2 Table). Therefore, we selected variables based on correlations. Firstly, we calculated the Pearson correlation coefficients between all environmental variables (including bioclimatic and topographic variables) (see S2 Table). Secondly, we standardized all environmental variables and used generalized linear models to calculate the standardized regression coefficients of the 22 variables for each vegetation type (see S3 Table). These standardized regression coefficients were used to compare the relative importance of each variable for predicting vegetation distribution. Thirdly, if the correlation coefficient between two variables was greater than 0.7 [50], we kept the one with stronger influences on vegetation distributions based on the regression coefficient. Finally, six climate variables and two topographic variables were selected: temperature seasonality (TS) (i.e. Bio4), precipitation seasonality (PS) (i.e. Bio15), mean temperature of warmest quarter (MTWQ) (i.e. Bio10) and the coldest quarter (MTCQ) (i.e. Bio11), precipitation of the warmest quarter (PWQ) (i.e. Bio18) and the coldest quarter (PCQ) (i.e. Bio19), slope and aspect. The selected variables are biological meaningful and have been widely used in previous studies on species distribution models [51–53]. TS and PS represent climate seasonality. MTWQ and MTCQ represent environment energy. PWQ and PCQ represent water availability. Aspect can reflect the solar radiation and slope reflects habitat heterogeneity [51]. These eight variables were used for the following model calibration. In order to directly compare the importance of each variable by means of the absolute coefficient values, the environmental variables were standardized.

We chose 80% of the present distribution data of each vegetation type randomly for model calibration and the remaining 20% for model evaluation. The area under the receiver operating characteristic curve (AUC) [54] was used to evaluate the performances of GLM and MaxEnt models. In general, the AUC values range from 0 to 1, with values close to 1 indicating near perfect model performance, values around 0.5 indicating random fit, and values below 0.5 indicating a tendency towards systematically wrong predictions.

The models based on both GLM and MaxEnt were used to predict the modern distribution of each vegetation type at a spatial resolution of 2.5 × 2.5 arc minutes. In order to account for the uncertainty caused by different model approaches (GLM, MaxEnt), the suitability of each vegetation type was calculated as the average of the outputs of the two models weighted by their AUC values [22, 55]. For each grid cell, the predicted suitability values of all vegetation types were compared and the vegetation type with the highest suitability was assigned as the most likely vegetation of that grid cell. In China, croplands, urban areas, planted forests and azonal vegetation occupy large regions and the natural vegetation in these regions is scarce. Therefore, our models predicted the potential natural distribution of vegetation in these areas. We calculated the range filling of each vegetation type as the ratio between the observed and predicted modern distribution [56], and used it to evaluate the influence of human activities on vegetation distribution.

Next, we used both the GLM and MaxEnt models to reconstruct vegetation distributions in the LGM and the MH. All models were fitted to the spatial resolution of 2.5 × 2.5 arc minutes. Similarly, the suitability map of each vegetation type was calculated as the average of the outputs of the two models weighted by their AUC values [22, 55]. For a grid cell, the vegetation type with the highest suitability was assigned as the most likely vegetation of that grid cell. To further evaluate the model reconstructions, we compared the modeled vegetation distributions with the reconstructed biomes based on pollen data. For each vegetation type, we used Kappa statistics to quantitatively evaluate the extent to which the results based on our models and pollen data matched with each other. To demonstrate the size changes of each vegetation type between different time periods, we calculated the proportional size of each vegetation type estimated as its area proportion to the terrestrial area of China based on modeled modern, the LGM and the MH vegetation distribution maps. We also estimated the percentage of persistence and changing areas of each vegetation type relatively to their past distributions between the LGM and the MH, and between the MH and the present. To understand how vegetation dispersed between different time periods due to climate changes, we calculated the centroids of each vegetation type in different time periods (the LGM, the MH, and the present), and demonstrated the direction of vegetation dispersal from the LGM to the MH, and from the MH to the present, and from the LGM to the present respectively.

MaxEnt [57] was conducted within the “dismo” package [58] of R. All other analyses were conducted in R version 3.1.1 [59].

## Results

Most AUC values of both GLM and MaxEnt models are above 0.9 (Table 1), which suggests that all models have high predictive power for the relationship between vegetation distribution and the environment as evidenced from an independent test dataset. The AUC values of the MaxEnt models were generally higher than those of the GLMs (Table 1), which suggests that the predictive power of MaxEnt is relatively better than that of GLM for most vegetation types. Compared to the observed modern vegetation distribution (Fig 1A), the modeled modern vegetation distribution (Fig 1B) maps potential natural vegetation in areas with heavy human disturbances. Specifically, temperate deciduous forests (veg 5), subtropical evergreen broadleaf forests (veg 6), alpine steppe (veg 16), grass and forb meadow (veg 17) and armoise and miscellaneous alpine meadow (veg 18) were predicted to occupy large proportions of modern urban areas, managed forest and agricultural areas, and have low range filling (Fig 1C).

**Table 1:** The AUC values of the Generalized Linear Models (GLMs) and MaxEnt for each vegetation type.

code	Vegetation type	GLM AUC	MaxEnt AUC
**veg 1**	taiga forest	0.896	0.954
**veg 2**	temperate coniferous forest	0.878	0.973
**veg 3**	tropical and subtropical mountain coniferous forest	0.941	0.966
**veg 4**	temperate coniferous and deciduous broadleaf mixed forest	0.97	0.99
**veg 5**	temperate deciduous broadleaf forest	0.882	0.902
**veg 6**	subtropical evergreen broadleaf forest	0.985	0.969
**veg 7**	tropical rainforest and seasonal rainforest	0.999	0.996
**veg 8**	temperate deciduous shrubland	0.864	0.923
**veg 9**	subalpine deciduous broadleaf shruband	0.909	0.926
**veg 10**	dwarf trees desert	0.939	0.975
**veg 11**	shrub desert	0.956	0.903
**veg 12**	alpine desert	0.97	0.978
**veg 13**	temperate steppe	0.841	0.957
**veg 14**	temperate bunch grass prairie	0.779	0.911
**veg 15**	desert steppe	0.815	0.929
**veg 16**	alpine steppe	0.918	0.921
**veg 17**	grass and forb meadow	0.787	0.857
**veg 18**	Alpine *Kobresia* spp. and forb meadow	0.877	0.889
**veg 19**	cool and temperate bog	0.926	0.967
**veg 20**	alpine tundra	0.939	0.934

### The driving factors of vegetation distribution

Our analyses indicated that mean temperature of the coldest quarter (MTCQ) and mean temperature of the warmest quarter (MTWQ) accounts for more variation in vegetation distributions than any other environment variables (S2 and S3 Tables). The vegetation types determined by MTCQ are mainly distributed in Northeast and Southwest China. In contrast, MTWQ dominates the distributions of tropical and subtropical coniferous forests in Southeast China and those of the alpine grassland on the Tibetan Plateau.

### Vegetation distribution during the Last Glacial Maximum

**Fig 2:**
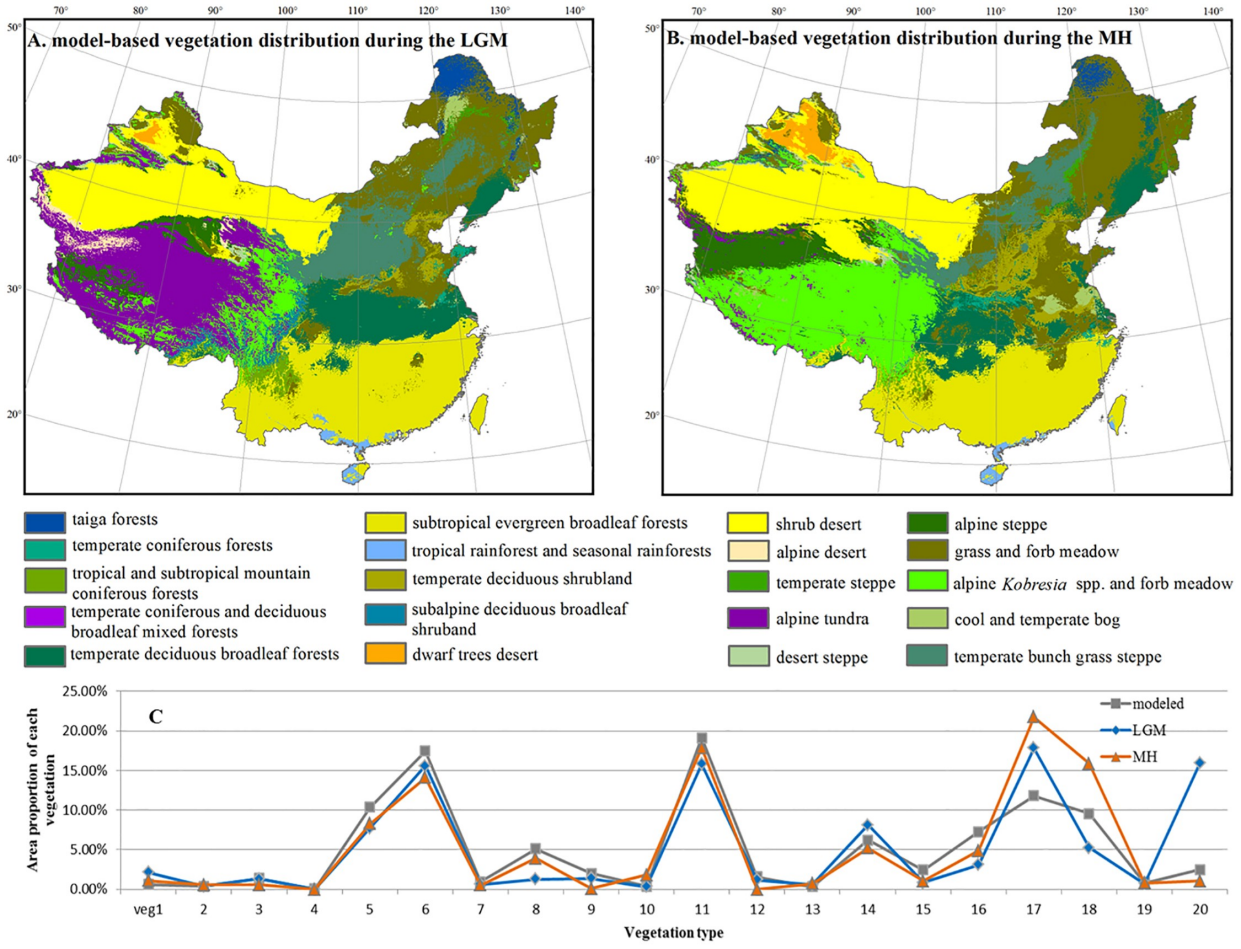
Hindcasted past distribution of natural vegetation during (A) the Last Glacial Maximum (LGM) and (B) the Mid-Holocene (MH) and (C) the proportion (%) of each vegetation type (LGM: dark blue line; MH: orange line; modeled: dark gray line). In (C), the proportion (%) of a vegetation type was calculated as the ratio of its distribution range relative to the terrestrial area of China.

**Fig 3:**
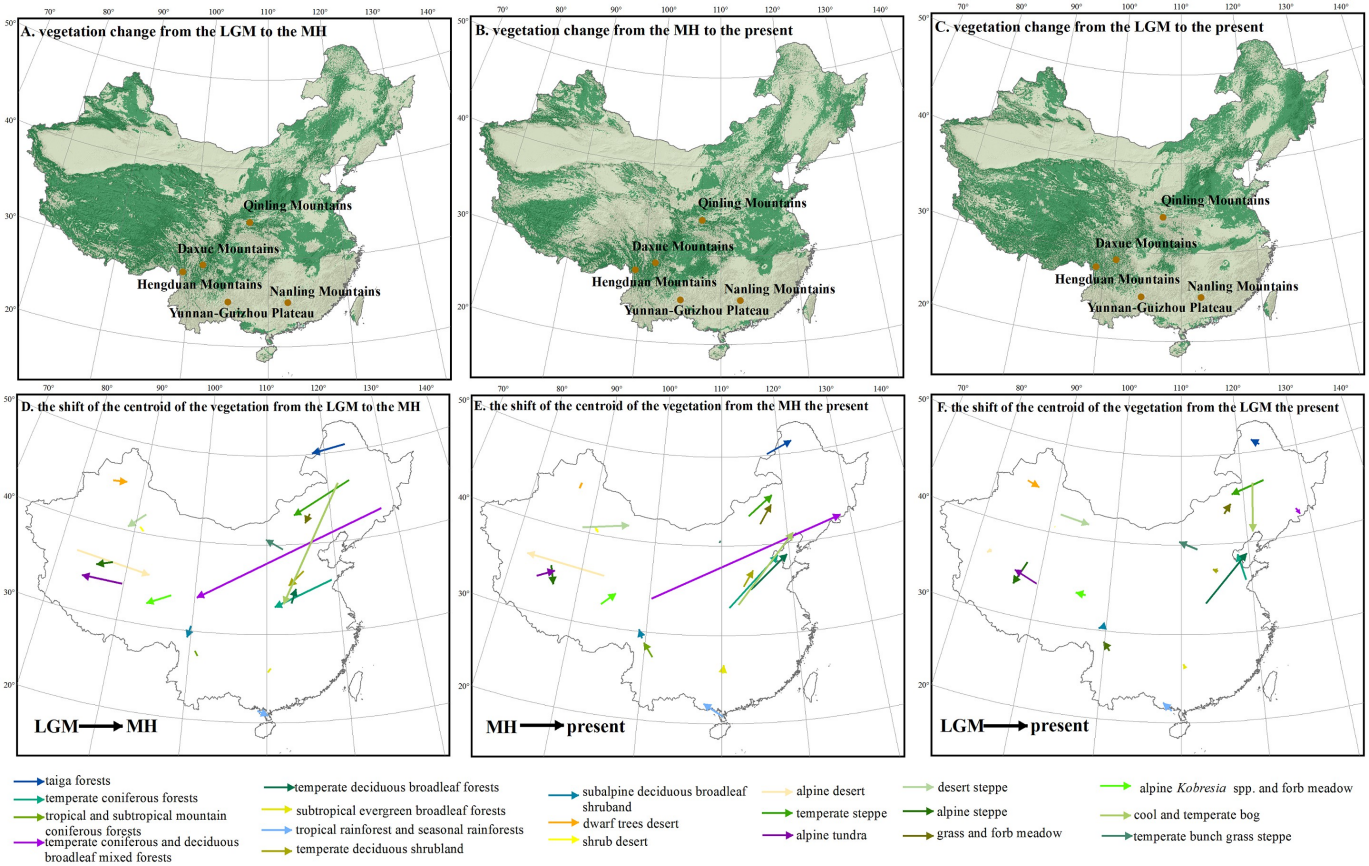
The regions with vegetation changes (dark green, A, B, C) and the dispersal directions of each vegetation type (D, E, F) from (A, D)the Last Glacial Maximum (LGM) to the Mid-Holocene (MH), (B, E) from the MH to the present and (C, F) from the LGM to the present.

The reconstructed vegetation maps of the LGM and MH are shown in Fig 2. Most vegetation types are hindcasted to have moved from northeast to the southwest from the LGM to the MH (Fig 3D), and moved back from the southwest to the northeast from the MH to the present (Fig 3E). Taken together, our results reveal relatively more northward distributions of most vegetation types in eastern China from the LGM to the present (Fig 3F).

**Fig 4:**
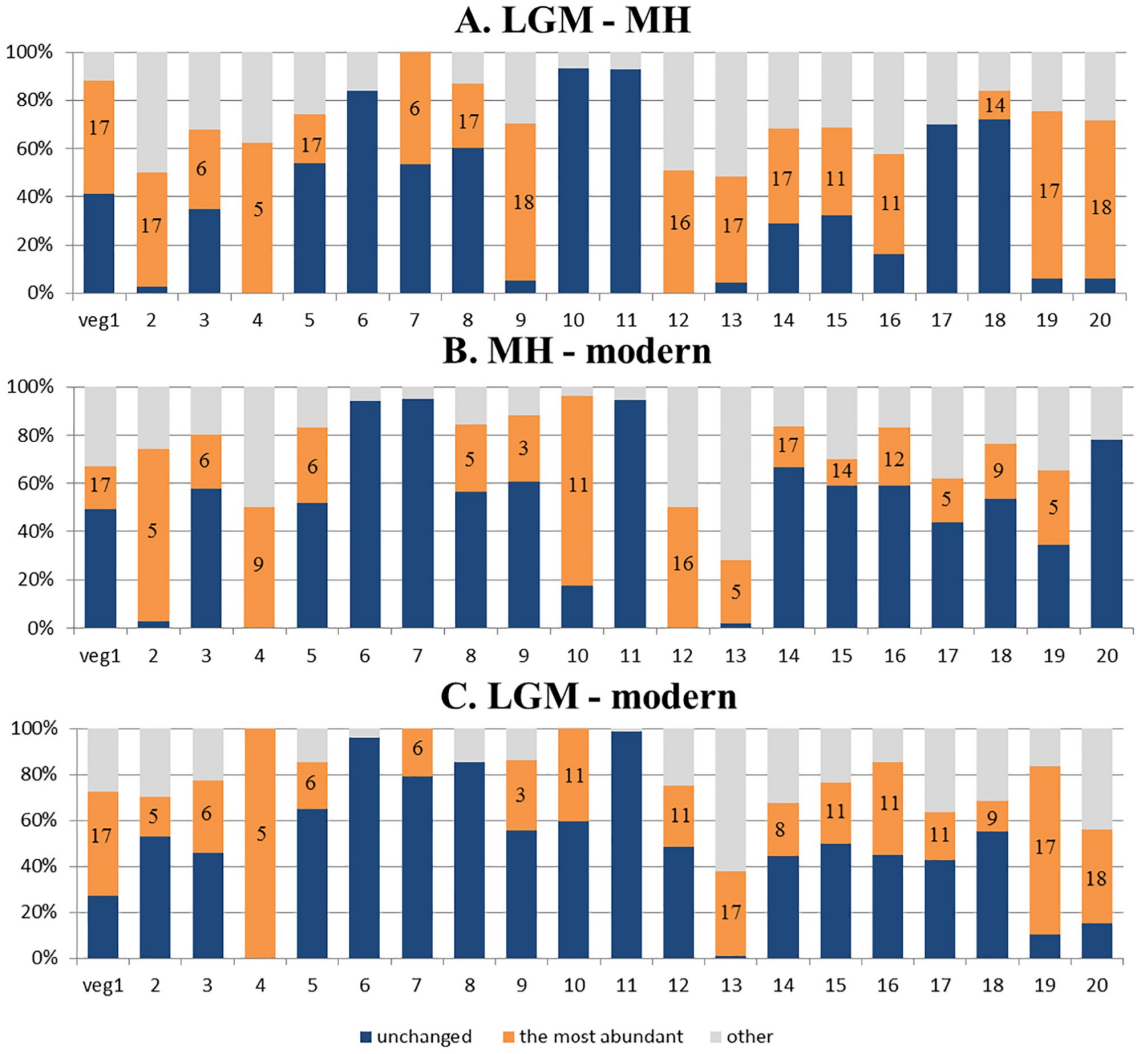
Percentage of persistence and changing vegetation distribution (A) from the Last Glacial Maximum (LGM) to the Mid-Holocene (MH), (B) from the MH to the present, and (C) from the LGM to the present. Dark blue: percentage of a vegetation type that remained unchanged between two time periods. Orange: percentage of a vegetation type that was converted to the most abundant new vegetation type (indicated with the type number in the orange bar). Grey: percentage of a vegetation type that was converted to all other vegetation types. For example, 40% of the LGM distribution range of veg 1 (taiga forest) remained unchanged at the present, 49% was converted to veg 17 (grass and forb meadow) in the MH and 11% were converted to other vegetation types (C).

The area proportions of the vegetation located in Southeast and Northwest China show little changes from the past to the present (Fig 2C). However, the Tibetan Plateau, North and Northeast China, and the areas near the 30°N in Central and Southeast China have experienced the highest turnover in vegetation due to climate change from the LGM to the MH and to the present (Fig 3). Specifically, in Northeast China, from the LGM to the MH, Grass and forb meadow (veg 17) expanded in North and Northeast China: 47% of temperate coniferous forest (veg 2), 44% of temperate steppe (veg 13) and 39% of temperate bunch grass prairie (veg 14) were transformed to grass and forb meadow (Fig 4A). From the LGM to the present, the coniferous forests shrunk substantially: 17% of temperate coniferous forests (veg 2) and almost all temperate coniferous and deciduous broadleaf mixed forests (veg 4) were transformed to temperate deciduous forests (veg 5) (Fig 4C). Compared to the LGM distributions, the south boundaries of taiga forests (veg 1) and temperate coniferous forests (veg 2) are both found more northward under current climate (see b in S1 Fig).

In North China, the eastern boundary of steppe vegetation types (veg 13 and veg 14) moved substantially westwards, and grass and forb meadow (veg 17) shrunk with its south boundary moving much northward from the LGM to the present. In contrast, the range of temperate deciduous forests (veg 5) expanded both eastward and northward at the cost of coniferous forests and temperate steppe. In Central and eastern China, the area of steppe has been reduced since the LGM, which are now covered by temperate deciduous forests (veg 5). Temperate deciduous shrublands (veg 8) occupied much larger area in the present than in the LGM.

On the Tibetan Plateau, the vegetation also shows substantial turnover from the LGM through the MH to the present. In the LGM, alpine tundra (veg 20) occupied the most area on the Tibetan Plateau, and was replaced by sagebrush and miscellaneous alpine meadow (veg 18) in the MH (Fig 2A and 2B). However, sagebrush and miscellaneous alpine meadows shrunk again towards the present.

### Vegetation distribution during the Mid-Holocene

In general, vegetation distributions moved northward from the MH to the present (Fig 3E). However, vegetation distributions responded to climate changes differently across space. The areas where the vegetation has been most strongly affected by climate change since the MH is consistent to those since the LGM (Fig 3B), including the Tibetan Plateau, North and Northeast China, and the areas near the 30°N in Central and Southeast China.

In North and Northeast China, from the MH to the present, temperate deciduous forests (veg 5) were shifted northward and expanded at the cost of 28% of the distribution of temperate deciduous shrublands (veg 8) and of 18% of grass and forb meadow (veg 17) (Fig 4B). In contrast, taiga forests (veg 1) in Northeast China shrunk slightly.

On the Tibetan Plateau, grass meadows and alpine tundra all occur more southward in the present than during the MH. Alpine steppes (veg 16) expanded from the MH to the present and replaced Alpine *Kobresia* spp. and forb meadow (veg 18) in many regions. Alpine tundra (veg 20) nearly disappeared in the MH, but occupies much larger areas in the present. Over 78% of dwarf tree deserts (veg 10) were replaced by desert shrublands (veg 11) and 50% of alpine deserts (veg 12) were replaced by steppes (veg 16) from the MH to the present (Fig 4B).

In the mountainous regions in the southeastern Tibetan Plateau, the tropical and subtropical mountain coniferous forests (veg 3) were greatly fragmented in the MH, but now expand in Hengduan Mountains (see c in S2 Fig). Besides, in eastern China, temperate coniferous forests (veg 2) disappeared during MH, but is now distributed in whole Shandong Peninsula (see b in S2 Fig).

### Model-based vs. pollen-based vegetation reconstruction

**Fig 5:**
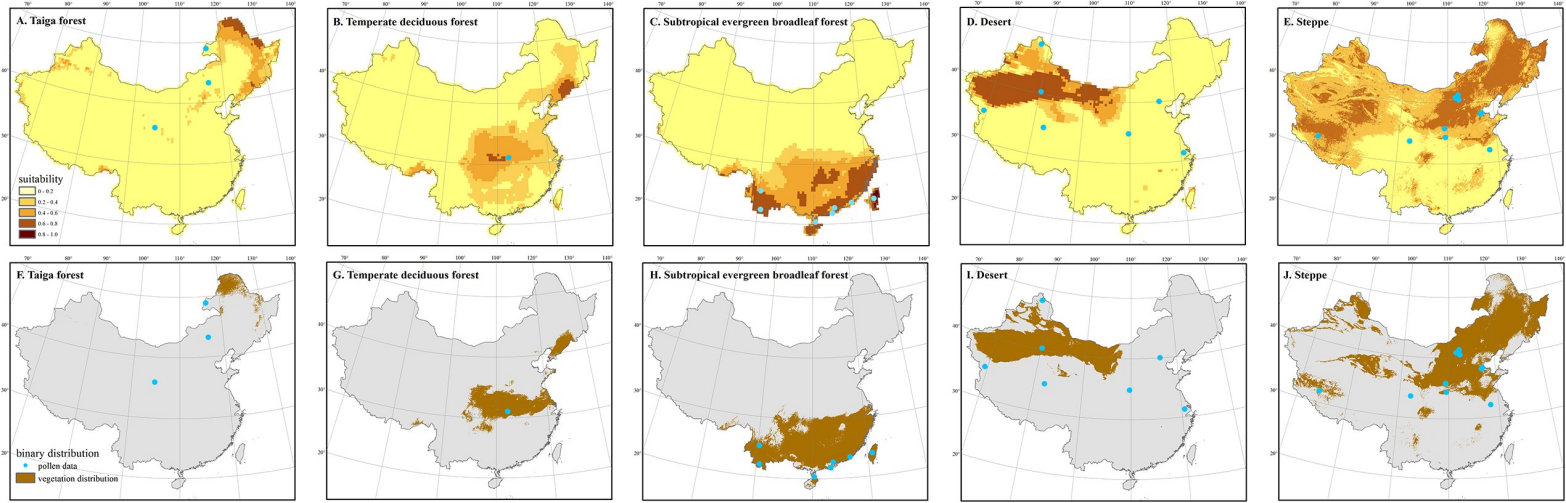
Comparison between model-based and pollen-based vegetation reconstructions during the Last Glacial Maximum (LGM). Panels (A) to (E) map the hindcasted climate suitability of the shown vegetation types, while panels (F) to (J) map the hindcasted binary distribution of each vegetation type. The blue dots in the maps represent the pollen sites for which the same vegetation type was identified during the LGM.

**Fig 6:**
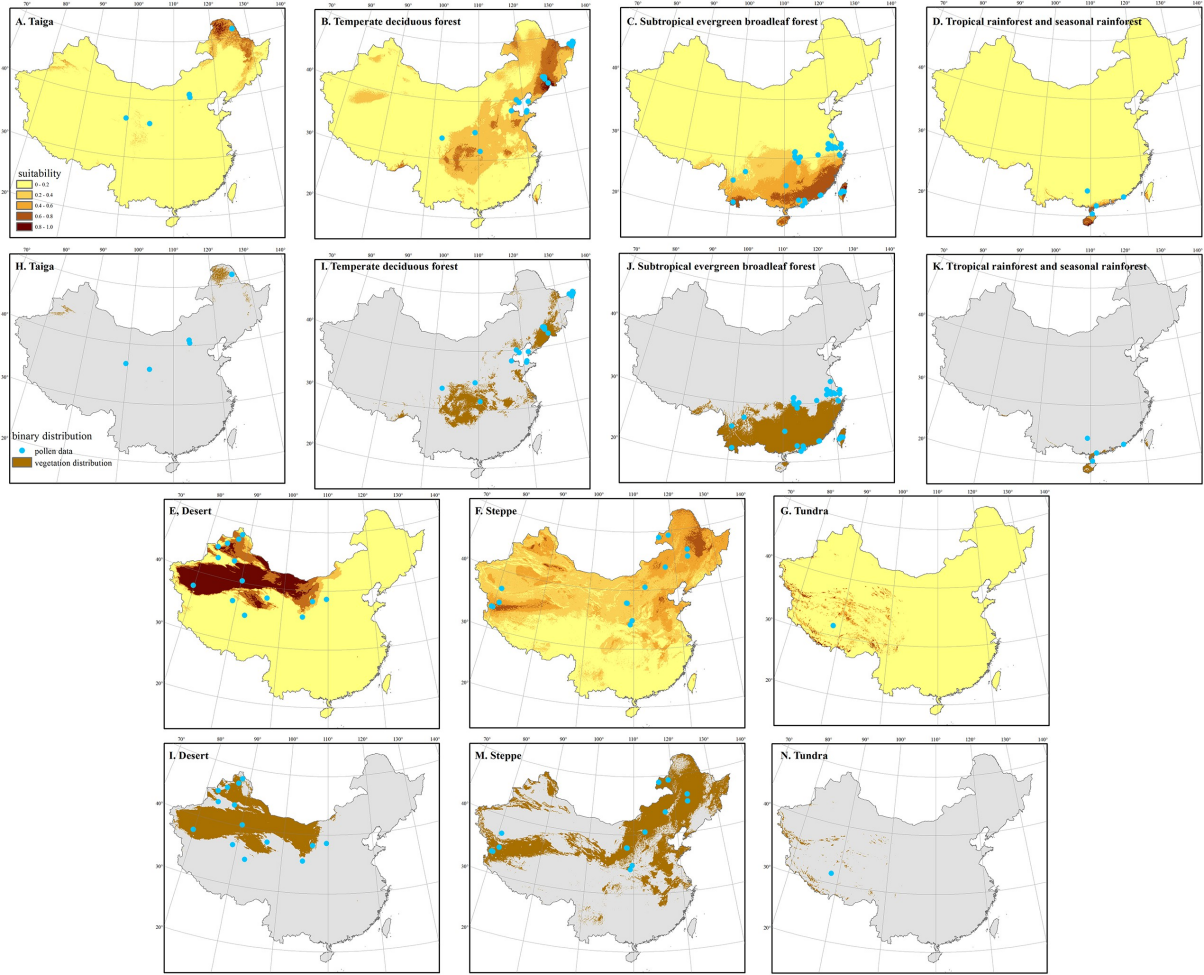
Comparison between model-based and pollen-based vegetation reconstructions during the Mid-Holocene (MH). Panels (A) to (G) map the hindcasted climatic suitability of the shown vegetation types, while panels (H) to (N) map the hindcasted binary distribution of each vegetation type. The blue dots in the maps represent the pollen sites for which the same vegetation type was identified for the MH.

To evaluate the performance of SDMs in reconstructing the past vegetation distributions, we compared the model-based vegetation with those reconstructed from pollen data [17, 34] (Figs 5 and 6) and calculated the Kappa value for each vegetation type (Table 2).

**Table 2:** Kappa values between model-based and pollen-based vegetation reconstructions in the LGM and the MH for seven major vegetation types in both LGM and MH.

Vegetation types	Kappa (LGM)	Kappa (MH)
**Taiga**	0.00	0.28
**Temperate deciduous forests**	0.47	0.46
**Subtropical evergreen forests**	0.90	0.64
**Desert**	0.37	0.73
**Steppe**	0.55	0.25
**Rainforest**	/	0.31
**Tundra**	/	0.50

In general, the vegetation reconstructed by pollen data is consistent with our results based on SDMs. In most cases, the pollen profiles for which a vegetation type is associated are located within or in very close distance to the modeled distribution range of the vegetation type. Most Kappa values for the comparison between modeled and pollen-based vegetation distributions are > 0.4 (Table 2), which suggests that the results of these two methods are in moderate agreement with each other [60]. For example, the distributions of subtropical evergreen forests reconstructed by our models and pollen data are highly consistent with each other during the LGM (Kappa = 0.90) and the MH (Kappa = 0.64) (Table 2). The temperate steppe distribution based on pollen data also generally agree very well with the modeled distribution during the LGM (Kappa = 0.55) (Table 2; Fig 5E), although it shows slight expansion to more southern regions than the modeled distribution during the MH (Kappa = 0.25). Similarly, the modeled distributions of deserts and tundra in the MH are also highly consistent with those based on pollen reconstructions (Desert: Kappa = 0.73; Tundra: Kappa = 0.50). The distribution of temperate deciduous forests reconstructed by our models (see e in S2 Fig) is moderately in line with the pollen-based reconstructions. Both methods supports a more southern distribution for this vegetation type during the LGM than during the MH and today [17, 34].

Although the model-based reconstructions and the pollen data were generally consistent with each other for most vegetation types tested, differences exist for some vegetation types. For example, the distribution of taiga forests based on pollen data is much more southward and westward than that of our prediction (see Figs 5A and 6A). The modeled distribution of deserts during the LGM differs from the locations of pollen sites [13, 17, 34, 61]. Specifically, pollen data suggest that temperate deserts had reached the present-day coastline in eastern China during the LGM [17, 34], while the modeled distribution map reveals much less expansion in desert area (see j-l in S1 Fig). During the MH, pollen data suggest larger desert distribution than today [17, 34], while the model-based distribution only showed marginal shifts (Fig 6 and S2 Fig).

## Discussion

### Major driving factors of vegetation distribution

Climate varies along elevation and latitudinal gradients, leading to changes in vegetation [62–64]. Ecologists have paid much attention to the major climatic drivers determining vegetation distribution for a long time [65, 66]. Fang and Yoda [23] studied the vegetation distribution in China systematically based on the Kira vegetation-climate classification system and delimited the range of temperature and precipitation of 29 vegetation types in China. Our study has revealed that the driving factors of vegetation distribution of vegetation are, to some extent, different. In general, we found that the influences of temperature on vegetation was stronger than that of precipitation [67]. Mean temperature of the coldest quarter had the strongest influence on vegetation distributions of forests in North and Northeast China (e.g. subalpine deciduous broadleaf shruband, alpine desert, desert steppe and grass and forb meadow, see S4 Table), which is in accordance with previous studies on the explanation of species richness patterns [52] and supports the frost-tolerance hypothesis [68].

It has been shown previously that human activities, such as over grazing, logging and land use, have strong direct impacts on vegetation and ecosystems [69, 70]. Consistent with previous findings, our results suggest that the vegetation (e.g. temperate deciduous broadleaf forest (veg 5), subtropical evergreen broadleaf forest and tropical rainforest (veg 6) and seasonal rainforest (veg 7)) in low altitude areas have been affected strongly by human activities (Fig 1C) [71]. China has a long history of highly-intensive agricultural activities through which the natural vegetation in low altitude areas, e.g. south-eastern China, has been destroyed and replaced by agricultural fields, settlements, and secondary vegetation. Our results demonstrate that the natural vegetation in this area would primarily consist of temperate deciduous forests (veg 5), subtropical evergreen broadleaf forests (veg 6), temperate deciduous shrublands (veg 8) and temperate bunch grass steppe vegetation (veg 14).

### Differences between vegetation reconstructions based on SDMs and pollen data

The spatial distributions of deserts, grasslands and shrublands in northern China since the LGM have been discussed controversially in previous studies. For example, early studies based on pollen records from the Loess Plateau of China indicated that the Loess Plateau was dominated by temperate steppe in the LGM [72], which is consistent with our findings. In contrast, studies based on plant functional types (PFT) derived from pollen records [17,34] suggested that desert expanded further east across the Loess Plateau and reached the eastern part of northern China during the LGM. A meta-analysis reconstructed an intermediate result and suggested that northern China was covered by semi-desert at that time [73]. These inconsistencies in the reconstruction of desert distribution in previous and the current studies could be partly due to differences in the definition of desert. In our analysis, vegetation type was based on *Vegetation Atlas of China* [38], in which sparse grasslands and shrublands are not categorized as desert. On the contrary, other studies [17, 34, 72] have considered sparse grassland and shrubland vegetation as deserts or semi-deserts. Also, since pollen can easily disperse by wind or water over very large distances, the location where pollen is found may not be the place of its production [74]. Finally, the precipitation reconstructions are known to be less precise for LGM and MH than temperature reconstruction, and may be another source of uncertainties in the model-based reconstruction of specifically drought- adapted vegetation types.

Lake sediment cores are the main type of pollen sampling in paleoecological studies in China. Lakes basins are open to air-borne influx of pollen from large distances, and the water is collected from equally large, if not even larger, regions and also from mountain snow [75]. It is therefore possible that pollen samples of lake sediments at lowlands may, to some extent, contain pollens from the highlands or from far away. This is especially true for *Picea*, *Pinus* and other needle-leaved tree pollen, which are abundantly found in lowland sediments [76] although they are normally the dominant species in mountain coniferous forests. This may explain why the reconstruction of taiga from pollen data was indicated to extend further south and west in contrast to our model predictions.

Although previous findings and our results suggest that even though the two methods both have uncertainties, SDMs and pollen data can, nevertheless, complement each other in the studies on vegetation dynamics under changing climate.

### Distribution of temperate forest in eastern Asia in the LGM

The distribution of temperate forests in eastern Asia during the LGM has long been debated. According to Qian and Ricklefs [77], temperate forests extended in eastern China and across the continental shelf to link populations in Korea and Japan, whereas Harrison et al. [78], based on a reconstructed vegetation map of eastern Asia, hypothesized that temperate forests had only a restricted distribution during the LGM. Our results support the latter view. Based on our findings, temperate deciduous forests had a smaller spatial extent in China during the LGM compared to the present distribution of this vegetation type (see e in S1 Fig) and were replaced by temperate steppe vegetation in several regions (Fig 3A).

### Mountain refugia in China

Mountains generally provide relatively more stable climate for organism than lowlands, as organisms in mountains are able to trace changing climates more easily within short distance [79]. Therefore, mountains act as refugia during periods of climate change, and offer shelter to many species, especially to small-ranged and endemic species [80]. Our results showed that the mountains in South and Central China, such as the Nanling Mountains, the Yunnan-Guizhou Plateau and the Qinling Mountains, encountered much less changes in the distributions of major vegetation types between the past and the present than other regions, which is consistent with previous findings [81]. Similarly, Qiu et al. [82] demonstrated with a pollen-based analysis that the vegetation in South China has been relatively stable since the Quaternary, which suggests that this region has possibly acted as refugia for vascular plants during the Quaternary. Such long stability may be one of the explanations for the extraordinary woody plant species diversity in this region [52]. In contrast, our results indicate a significant dynamics in the vegetation distribution in the Hengduan Mountains and Daxue Mountains in southwestern China. Therefore, it is quite likely that the changes in vegetation distribution may have significantly influenced the dispersal and hence the phylogeographical structure of plant species in these regions [83, 84]. Previous findings and our results suggest that biological refugia and species persistence may not be the dominant factor in shaping the species diversity in the Hengduan Mountains [85].

### Limitations of SDM for the prediction of vegetation distribution

Model-based analyses can complement pollen analyses for studying vegetation-climate relationships and the dynamics of vegetation distribution, yet the model results should be interpreted with caution. The expansion of vegetation in response to climate change is a long-term process. Recent studies have suggested that the response of vegetation distribution to climate change may have a time lag [86, 87]. The methods to take the time lag into consideration in SDM calibrations is challenging, but one way is to integrate SDMs with macroecological methods as shown by Araújo et al. [88] and Guisan & Rahbek [89]. The distribution of some vegetation types may not be in equilibrium with climate, which may also increase uncertainty in model calibration and prediction [90]. Moreover, recent studies suggest that different SDM algorithms and climate scenarios could also introduce uncertainties in the predictions of species and vegetation distribution [91].

Despite these limitations, the SDM-type approach is a useful tool to study the relationship between vegetation and climate. Compared to other methods, the vegetation distribution predicted by SDMs is quantitative and spatially explicit, and it can be applied to any typology of vegetation classification and is not restricted to major plant functional types as in dynamic vegetation models. The flexibility in the choice of environmental variables as predictors and statistical models allows for tuning the analysis to a range of research questions. Optimizing the statistical modeling in order to include information on relative climate stability and response lags under changing climate conditions will further improve the robustness of vegetation-climate models in projecting the distribution of vegetation under past and future conditions.

## Supporting information

S1 Fig Distribution changes of each vegetation type from the Late Glacial Maximum (LGM) to the present. Brown: distributions under both current and the LGM climates; light red: distributions during LGM; green: present distributions.
(TIF)

S2 Fig Distribution changes of each vegetation type from the Mid-Holocene (MH) to the present. Brown: distributions under both current and the MH climates; light red: distributions during the MH; green: present distributions.
(TIF)

S1 Table Environmental variables used in the analysis. (PDF)

S2 Table The correlation coefficient between all environmental variables. (PDF)

S3 Table Standardized regression coefficients of all 22 environmental variables considered to explain vegetation distribution estimated by generalized linear models. The highest number in each line indicates the variable that best explains the distribution of that vegetation type alone. See Table 1 for definition of vegetation and S1 Table for environmental factors.
(PDF)

S4 Table Standardized regression coefficients of the selected environmental variables in explaining vegetation distribution estimated by generalized linear models. The highest number in each line indicates the variable that best explains the distribution of that vegetation type alone. See Table 1 for definition of vegetation and S1 Table for environmental factors.
(PDF)
