# Supplementary figures and images for: Response of spatial vegetation distribution in China to climate changes since the Last Glacial Maximum (LGM)
Source: PLoS One. 2017 Apr 20;12(4):e0175742. doi: 10.1371/journal.pone.0175742 (PMC5398547; doi:10.1371/journal.pone.0175742)

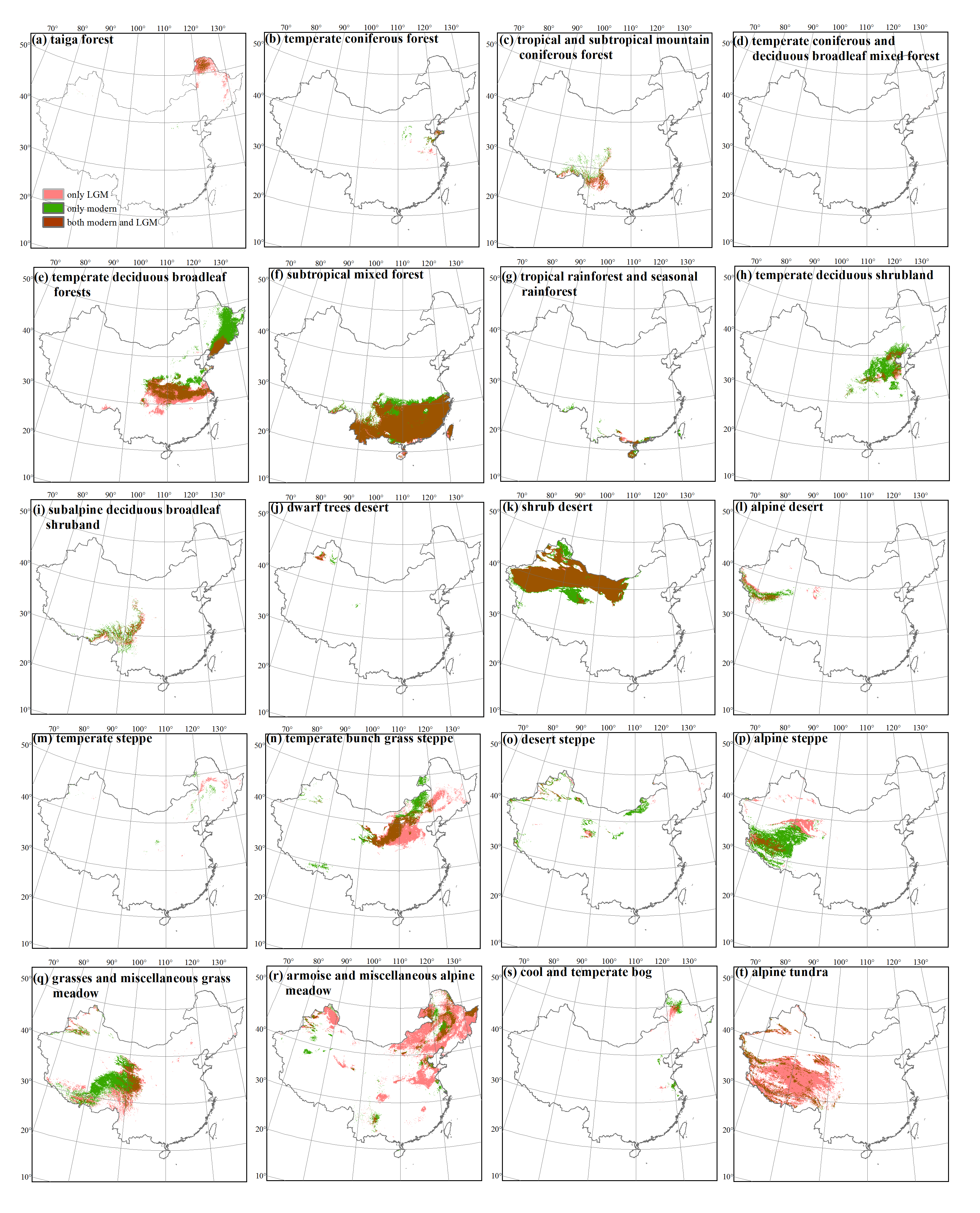

Supplement: S1 Fig — Brown: distributions under both current and the LGM climates; light red: distributions during LGM; green: present distributions. (TIF) [file pone.0175742.s001.tif]

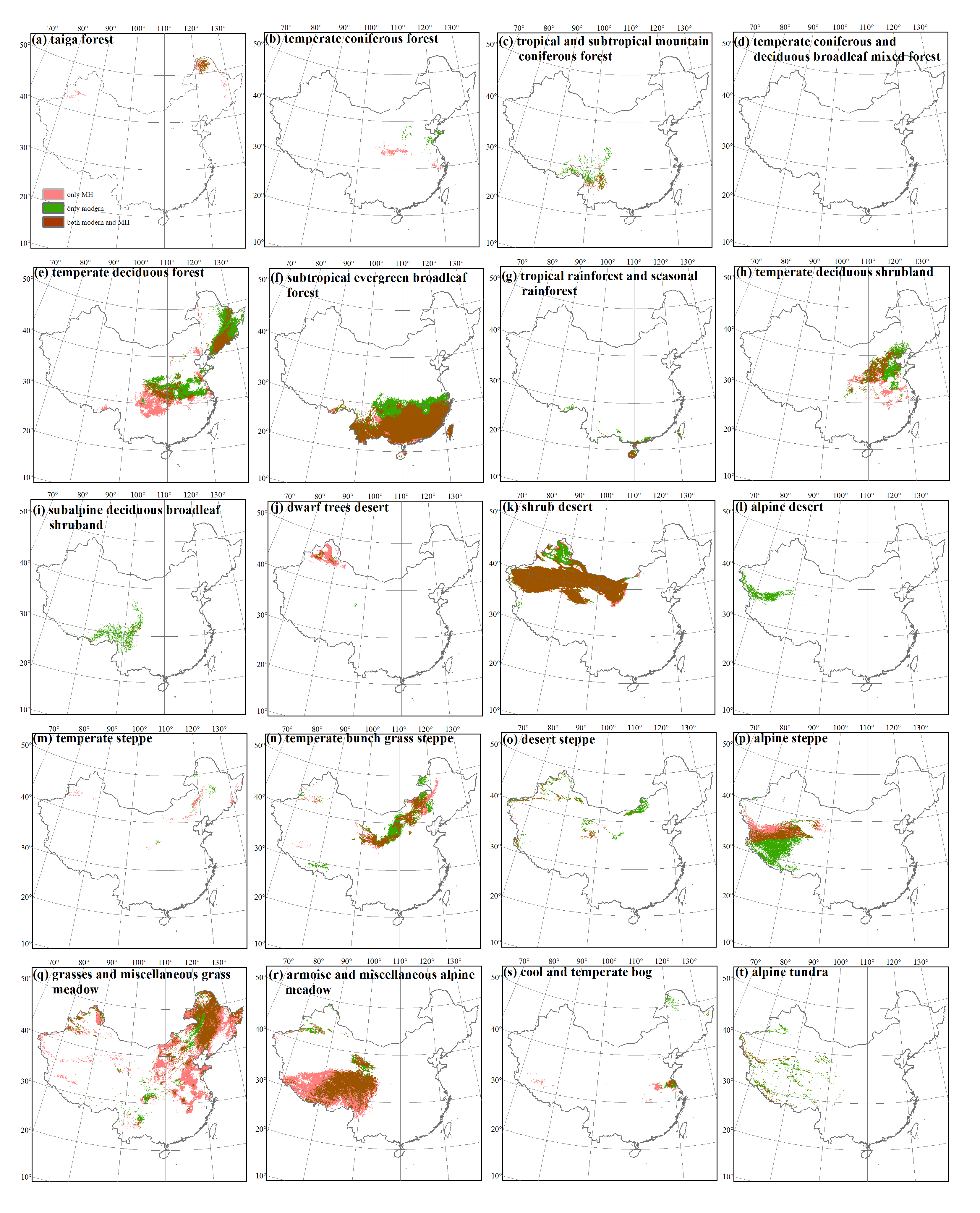

Supplement: S2 Fig — Brown: distributions under both current and the MH climates; light red: distributions during the MH; green: present distributions. (TIF) [file pone.0175742.s002.tif]
