# Supplementary material for: Response of spatial vegetation distribution in China to climate changes since the Last Glacial Maximum (LGM)
Source: PLoS One. 2017 Apr 20;12(4):e0175742. doi: 10.1371/journal.pone.0175742 (PMC5398547; doi:10.1371/journal.pone.0175742)
Supplement: S2 Table — (PDF) [file pone.0175742.s004.pdf]

S2 Table. The correlation coefficients between all environmental variables.

	bio1	bio2	bio3	bio4	bio5	bio6	bio7	bio8	bio9	bio10	bio11	bio12	bio13	bio14	bio15	bio16	bio17	bio18	bio19	alt	aspect	slope
**bio1**	1																					
**bio2**	-0.7	1																				
**bio3**	-0.07	0.29	1																			
**bio4**	-0.36	0.36	-0.75	1																		
**bio5**	0.8	-0.45	-0.53	0.25	1																	
**bio6**	0.92	-0.76	0.18	-0.67	0.53	1																
**bio7**	-0.5	0.57	-0.59	0.97	0.11	-0.79	1															
**bio8**	0.84	-0.49	-0.43	0.16	0.97	0.59	0.01	1														
**bio9**	0.9	-0.66	0.27	-0.7	0.5	0.98	-0.78	0.55	1													
**bio10**	0.88	-0.57	-0.46	0.12	0.98	0.64	-0.04	0.98	0.6	1												
**bio11**	0.92	-0.69	0.27	-0.7	0.5	0.99	-0.79	0.57	0.98	0.62	1											
**bio12**	0.72	-0.79	0.07	-0.54	0.36	0.8	-0.67	0.43	0.75	0.49	0.78	1										
**bio13**	0.65	-0.71	0.12	-0.5	0.31	0.72	-0.62	0.4	0.66	0.44	0.71	0.96	1									
**bio14**	0.67	-0.79	-0.19	-0.35	0.45	0.72	-0.51	0.43	0.67	0.54	0.67	0.85	0.72	1								
**bio15**	-0.58	0.65	0.27	0.2	-0.47	-0.57	0.32	-0.42	-0.55	-0.51	-0.52	-0.51	-0.33	-0.67	1							
**bio16**	0.67	-0.72	0.15	-0.53	0.3	0.75	-0.65	0.4	0.69	0.44	0.74	0.97	0.99	0.73	-0.36	1						
**bio17**	0.66	-0.79	-0.16	-0.37	0.43	0.72	-0.52	0.41	0.68	0.53	0.67	0.85	0.72	0.99	-0.67	0.73	1					
**bio18**	0.63	-0.67	0.19	-0.53	0.26	0.7	-0.64	0.38	0.65	0.39	0.7	0.94	0.98	0.64	-0.3	0.98	0.64	1				
**bio19**	0.63	-0.75	-0.13	-0.37	0.4	0.69	-0.52	0.37	0.66	0.49	0.65	0.83	0.71	0.97	-0.65	0.72	0.98	0.62	1			
**altitude**	-0.69	0.5	0.64	-0.38	-0.93	-0.41	-0.2	-0.93	-0.33	-0.93	-0.37	-0.41	-0.4	-0.45	0.41	-0.38	-0.43	-0.35	-0.39	1		
**aspect**	0.03	-0.07	0	-0.04	-0.01	0.04	-0.06	0.01	0.03	0.01	0.04	0.07	0.07	0.04	-0.03	0.07	0.04	0.08	0.03	-0.01	1	
**slope**	-0.03	0	0.09	-0.08	-0.08	0.01	-0.07	-0.07	0.02	-0.07	0.02	0.02	0.02	-0.01	-0.01	0.02	0	0.03	0	0.08	-0.4	1
