# Supplementary material for: Response of spatial vegetation distribution in China to climate changes since the Last Glacial Maximum (LGM)
Source: PLoS One. 2017 Apr 20;12(4):e0175742. doi: 10.1371/journal.pone.0175742 (PMC5398547; doi:10.1371/journal.pone.0175742)
Supplement: S4 Table — The highest number in each line indicates the variable that best explains the distribution of that vegetation type alone. See Table 1 for definition of vegetation and S1 Table for environmental factors. (PDF) [file pone.0175742.s006.pdf]

S4 Table. Standardized regression coefficients of the selected environmental variables in explaining vegetation distribution estimated by generalized linear models.

coefficient	veg1	veg2	veg3	veg4	veg5	veg6	veg7	veg8	veg9	veg10	veg11	veg12	veg13	veg14	veg15	veg16	veg17	veg18	veg19	veg20
**Intercept**	-4.69	-0.11	10.89	-4.31	-7.06	3.03	-18.54	-10.44	11.43	-10.07	-0.68	3.20	-12.23	-5.78	-3.20	1.81	-7.45	3.13	-15.95	3.21
**TS (bio4)**	0.00	0.00	-0.01	-0.03	-0.01	-0.01	-0.01	-0.02	0.00	-0.01	0.00	-0.02	-0.01	-0.01	-0.02	0.01	0.00	0.01	0.00	0.00
**MTWQ (bio10)**	-0.08	0.60	0.23	1.07	0.58	0.19	0.26	0.62	0.08	0.29	0.15	0.85	0.20	0.43	0.59	-0.28	-0.16	-0.45	-0.05	-0.12
**MTCQ (bio11)**	0.05	-0.57	-0.23	-1.11	-0.58	-0.17	-0.15	-0.60	-0.09	-0.28	-0.13	-0.89	-0.20	-0.43	-0.60	0.26	0.17	0.42	0.05	0.05
**PS (bio15)**	-0.05	0.01	-0.04	-0.13	-0.02	-0.04	-0.06	0.04	-0.05	-0.01	0.01	-0.01	0.03	0.02	0.00	0.02	0.00	-0.02	0.02	-0.02
**PWQ (bio18)**	0.01	0.00	0.00	0.02	0.00	0.00	0.00	0.00	0.01	-0.02	-0.02	-0.01	0.00	0.00	-0.01	-0.01	0.00	0.00	0.00	0.00
**PCQ (bio19)**	-0.05	0.00	-0.03	-0.16	0.00	0.01	-0.04	0.00	-0.06	0.02	0.01	-0.08	0.00	-0.05	0.02	0.02	-0.01	-0.02	0.00	-0.01
**aspect**	0.00	0.00	0.00	0.00	0.00	0.00	0.00	0.00	0.00	0.00	0.00	0.00	0.00	0.00	0.00	0.00	0.00	0.00	0.00	0.01
**slope**	0.00	0.01	0.00	0.00	0.00	0.00	0.00	0.00	0.00	0.00	0.00	0.00	0.00	0.00	0.00	0.00	0.00	0.00	0.00	0.01

The highest number per line indicates the variable that best explains the distribution of that vegetation type alone. See Table 1 for definition of vegetation and S1 Table for environmental factors
